# Effect of Temporomandibular Disorders on Quality of Life for Elderly Individuals

**Published:** 2018-10

**Authors:** Thomás SANTANA SÓRIA, Giordano SANTANA SÓRIA, Raul ANTÔNIO CRUZ, Myrian CAMARA BREW, Eduardo GROSSMANN, Caren SERRA BAVARESCO

**Affiliations:** 1. Dept. of Basic Health, City Hall of Porto Alegre, Porto Alegre, Rio Grande do Sul, Brazil; 2. Dept. of Epidemiology, Federal University of Pelotas, Pelotas, Rio Grande do Sul, Brazil; 3. Dept. Odontology, Luteran University of Brazil, Canoas, Rio Grande do Sul, Brazil; 4. Federal University of Rio Grande do Sul, Porto Alegre, Rio Grande do Sul, Brazil; 5. The Graduate Program in Dentistry, Luteran University of Brasil, Canoas, Rio Grande do Sul, Brazil

## Dear Editor-in-Chief

Temporomandibular Disorders (TMD) are characterized by a set of signs and symptoms that affect structures associated with the masticatory system, mainly the temporomandibular joint (TMJ) and the muscles involved in jaw movement (1). Since TMD are often associated with pain and functional impairment, assessments of oral health-related quality of life (ORQL) appear to be of fundamental importance in the presence of TMD. To obtain information regarding ORQL, several instruments have been developed, including the Geriatric Oral Health Assessment Index (GOHAI) (2). Thus, the present study aimed to evaluate the effect of TMD on the ORQL of elderly users of a basic health unit in the city of Porto Alegre, Rio Grande do Sul (RS), Brazil.

This investigation utilized a cross-sectional design with convenience sampling. Overall, 112 individuals of both genders over 60 yr of age, enrolled in the health unit and sought outpatient dental care from Jan to Oct 2014 were consecutively interviewed.

All individuals received and signed an informed consent. To evaluate signs and symptoms of TMD, the Fonseca Anamnestic Index was used.

This questionnaire includes 10 objective questions about TMD; for each question, the possible responses are yes (10 points), sometimes (5 points) and no (0 points). TMD was classified as absent (0 to 15 points), mild (20 to 40 points), moderate (45 to 65 points) or severe (70 to 100 points) (3). The GOHAI was used to assess quality of life-based on the elderly subjects’ self-perceived oral health. This index consists of 12 questions that involve analyzing information provided by respondents themselves regarding the effects of their oral health problems on physical, psychological and pain dimensions. From these answers, scores are generated and used to define the overall GOHAI score, which varies from 12 to 36 points. Higher scores indicate better self-perceived oral health-related quality of life (2). Based on their scores, specific exposure and outcome variables were treated as discrete numerical variables, dichotomous variables or categorical variables. Stata software (copyright 1985–2011, StataCorp LP) was used for tabulation and data analysis. Significance tests were performed to test the association between TMD and quality of life as well as correlations and interactions involving other variables.

The present study was approved by the Ethics Committee of Conceição Hospital Group (number 566553).

The study population consisted of 62.5% women and the most common age group and household income class were 65 to 69 yr of age and class B (41.4%), respectively. With respect to the degree of TMD severity, mild intensity was most common (40.7%); the mean TMD score for the sample was 29.9 points. Notably, the frequencies of other TMD intensities in this population were 33.6%, 18.6% and 7.1% for no TMD, moderate TMD and severe TMD, respectively. The mean GOHAI score was 25.2 points, and respondents predominantly exhibited low self-perceived oral health (84.9%). Based on the corresponding confidence intervals, gender, age and income were not statistically associated with GOHAI score (Table 1). The effect of TMD on ORQL was calculated via a linear regression analysis involving categorized TMD intensity and GOHAI score as a continuous variable. This analysis showed an association between TMD intensity and GOHAI score that gradually increases with level of exposure.

**Table 1:** Linear regression results (means and *P*-values) for GOHAI score

	***Mean GOHAI score***	**P-*value***
**Gender**		
Male	0.22 (−1.50:1.94)	0.801
Female	Ref	
**Age (yr)**		
75 or older	1.68 (−0.55:3.91)	
70 to 74 yr	1.44 (−1.28:4.15)	0.448
65 to 69 yr	0.53 (−1.54:2.61)	
60 to 64 yr	Ref	
**Household income (class)**		
A	1.83 (−1.75:5.42)	
B	1.78 (−1.07:4.63)	0.500
C	2.23 (−0.64:5.11)	
D/E	Ref	

This study showed an association between TMD intensity and individuals’ self-perceived oral health, demonstrating the impact of TMD-related issues on quality of life. The aforementioned self-perception results were not confirmed in a cohort study (4) conducted in Sweden with 6346 patients with 50–65 yr of age; a high proportion of elderly subjects were satisfied with their oral health status. Low satisfaction with oral health is related to low education level and that the promotion of healthy lifestyles and access to oral health care can increase satisfaction with oral health (4). Inconsistencies between that‘s results and our findings may be related to sample size, study design and differences in socioeconomic conditions between Brazil and Sweden. In this study, the interviewed elderly individuals were predominantly from families in class B (41.1%) or C (37.5%). The subjects had middle socioeconomic status and therefore had a relatively high education level, positively influenced their self-perceived oral health (4,5). Low overall GOHAI scores were obtained in this study, indicating a negative impact of oral health on the daily lives of the evaluated elderly subjects. It is necessary to have more qualified professionals conducting the diagnosis and treatment of TMD in elderly patients to improve these patients’ quality of life.
